# Chairman’s Communist Party of China member status and targeted poverty alleviation: Evidence from China

**DOI:** 10.1371/journal.pone.0284692

**Published:** 2023-06-15

**Authors:** Jian Xie, Ruirui Gu, Tianyi Lei, Sen Yang, Ruian Yu

**Affiliations:** 1 Department of International Accounting, School of International Education, Guangxi University of Finance and Economics, Nanning, China; 2 School of Management, Guangxi Minzu University, Nanning, China; 3 Department of Financial Engineering, School of Finance, Zhongnan University of Economics and Law, Wuhan, China; 4 Department of Business, Huanggang Normal University, Huanggang, China; 5 Department of Finance, School of Economics and Management, Hubei University of Technology, Wuhan, China; Newcastle University Business School, UNITED KINGDOM

## Abstract

Based on the data of Chinese listed private companies from 2016 to 2020, this paper investigates the influence of the Chairman’s member status of Communist Party of China (CPC) on targeted poverty alleviation. The research results demonstrate that the Chairman’s CPC member status of private companies significantly increases the companies’ willingness and the amounts of investment in poverty alleviation. The construction of the CPC organization can strengthen the role of the chairman’s Communist Party of China member status in promoting targeted poverty alleviation. The conclusions are still valid through robustness tests, such as substituting dependent variables, adjusting the sample range, and PSM-paired samples. In addition, the Impact Threshold for a Confounding Variable is used to deal with endogenous problems.

## 1. Introduction

Based on the upper echelon theory (Hambrick & Mason, 1984) [1], existing studies have found when the demographic background variable is taken as a proxy variable to describe the cognitive structure and values of managers, the certain personal characteristics of managers will affect the performance of corporate social responsibility. For example, demographic background characteristics like gender, age, education [2], and acquired experience like overseas experience [3], poverty experience [4], and so on.

The culture which is a deep-seated factor for managers to make differentiated and diversified social responsibility decisions affects managers’ cognition, interaction and strategy choices [5].Cultural innovation is emerging in the field of finance [6], in which the impact of managers’ cultural background characteristics on corporate social responsibility has also emerged as a hot research topic. China not only has traditional cultures such as Confucianism and Taoism, but also has an ideal of Communism that is gradually developing and radiating strong vitality. It enriches the research of ‘culture and corporate behavior’. Above mentioned two cultures both make companies assume more social responsibilities. Communist Party of China (CPC) membership of the managers is an important carrier for participating in political learning and inheriting the red culture of communism. Studies have shown that the party membership of managers will increase corporate charitable donations [7] and promote corporate social responsibility [8].

Poverty alleviation is a special social responsibility given to private companies in this era. It is similar to other social responsibilities such as environmental protection, charitable donations, stakeholder responsibility, and is also endowed with its special attributes. It aims to eliminate poverty and achieve common prosperity. Besides, it is also a necessary process for the realization of Communism and an inevitable choice for the Socialist system. The data shows among the A-share private listed companies from 2016 to 2020, up to 31.1% of the chairmen were members of the CPC. Besides, both the willingness to participate in targeted poverty alleviation and the amount of poverty alleviation investment in private companies with the status of the CPC member were significantly higher than the private companies to which the chairman of the non-CPC member belongs. The research data demonstrates that there should be some important internal connection between the CPC member status of the chairman of a private company and targeted poverty alleviation.

With the support of Chinese government, the private companies have become an important force for poverty alleviation. For one thing, when the private entrepreneurs are absorbed to become the CPC members, they need to learn systematic political knowledge such as Communist values and pass strict political review. For another, the establishment of grassroots organizations of CPC in the companies has easy access to the ideology of Communist values. Besides, the CPC is the leadership core for the cause of socialism with Chinese characteristics. The setup of grassroots organizations of CPC contributes to the closer relationship between the government and companies. Therefore, we reasonably expect that the chairman of the CPC member has more identify with Communist values and also prefers to cooperate with the government.

In this paper, we select Chinese listed private companies from 2016 to 2020 as samples to investigate the influence of chairman’s CPC membership of private companies on targeted poverty alleviation. The results find out that compared with private companies without CPC chairman status, the private companies with the chairman status of the CPC membership are significantly more willing to participate in targeted poverty alleviation and invest much more in poverty alleviation. Besides, the role of the chairman’s CPC member status in promoting targeted poverty alleviation depends on the construction of the Party organization. In other words, the chairman’s CPC member status has a more significant impact on targeted poverty alleviation in those private companies with grassroots Party organizations.

The main contributions of this paper are as follows. First, it has enriched the research on the influencing factors of poverty alleviation in Chinese listed companies. Existing research mainly studies the influencing factors of targeted poverty alleviation from other aspects of companies, while this paper finds that private company executive politics identity is also an important factor in targeted poverty alleviation, and it is a useful supplement to this field. Second, it expands the research on the upper echelon theory in China. Based on the perspective of Communist culture, this paper uses the managerial CPC membership to characterize its cultural background characteristics, investigates its impact on targeted poverty alleviation, and expands the relevant research on the contextualization of the upper echelon theory in China. Third, this paper investigates the impact of senior management political membership on the targeted poverty alleviation of private companies, and has important enlightenment for how to effectively connect poverty alleviation and rural revitalization, and how to guide private companies to participate in poverty alleviation during the rural revitalization stage.

## 2. Institutional background and hypothesis development

### 2.1. Institutional background

#### 2.1.1. Private listed companies’ participation in targeted poverty alleviation

The history of human civilization is a history of the struggle against poverty. Since its inception, the People’s Republic of China has been committed to poverty governance and has made many remarkable achievements. China’s new generation of leaders has placed particular emphasis on this work. On November 3, 2013, President Xi Jinping first proposed ‘targeted poverty alleviation’ in a visit to the Eighteenth Cave Village in Huayuan Town, Hunan Province. He stated that poverty alleviation should be practical and tailored to local conditions. In November 2015, President Xi Jinping made an important speech and pledged to completely eliminate poverty by 2020, which was called ‘Commitment 2020’. In order to complete this achievement, with the guidance of the CPC, the community has responded extensively, gradually forming a major pattern of poverty alleviation with the participation of many parties.

Private companies are essential parts of the Socialist market economy with Chinese characteristics and play as important living forces in combating poverty. On 17 October 2015, the All-China Federation of Industry and Commerce, the State Council Poverty Alleviation Office and the China Society for Promotion of the Guangcai Program launched the ‘Ten Thousand Enterprises Helping Ten Thousand Villages’ program. The program took private companies as the major sponsors, with the financially disadvantaged villages and households established as the objects of financial aid. It focused on bridging village and companies, and mobilizing more than 10,000 private companies nationwide to help more than 10,000 poor villages to accelerate the process of poverty eradication. It aimed to promote all-round healthy growth of non-public economy and build a moderately prosperous society on all fronts within 3 to 5 years.

Furthermore, aiming to guide and encourage capitals to flow in targeted poverty alleviation, in September 2016, China Securities Regulatory Commission encouraged listed companies to fulfill their social responsibility of targeted poverty alleviation. This served the national poverty alleviation for the interest of the national overall strategy. Subsequently, Chinese Stock Exchange unequivocally requires listed companies to reveal information on targeted poverty alleviation, including four aspects of targeted poverty alleviation planning, an annual summary of targeted poverty alleviation, the effectiveness of targeted poverty alleviation and follow-up precise poverty alleviation plans in the social responsibility in their annual reports.

#### 2.1.2. Party-building system in private companies

With the reform and opening up policy, the private economy has achieved historical development from scratch, from small to large, from weak to strong, and grown into an important part of the socialist market economy. Moreover, the private companies have undergone an evolutionary process from a ‘dissident force’ in socialism to an ‘important basis’ for China’s economic development, and private entrepreneurs are ‘their own people’. Private companies have also undergone an evolutionary process of deepening, improving and maturing from a socialist ‘dissident force’ to an ‘important foundation’ for China’s development. On one hand the strengthening of the Party-building work of private companies could guide and regulate the development of the private economy. On the other hand, it is also an inherent requirement for the overall strengthening of the Party leadership.

The work of the Party-building in private companies mainly includes two aspects of political absorption and the Party organization building. The Party organization of private companies is a unique product of the socialist system, and by embedding grassroots Party organizations in private companies. It achieves the penetration of the CPC’s influence within private companies, thus ensuring its leadership of the private economy.

### 2.2. The upper echelon theory and corporate social responsibility

The upper echelon theory was proposed by Hambrick and Mason [9] in 1984, which regarded managers as heterogeneous economic people with bounded rationality. It uses demographic characteristics such as gender, age, and educational background to portray the differences in the cognitive structure and values of managers, and believes that these differences will affect the behavior of companies. The proposal of this theory pushes the research on the influence of managers’ characteristics on corporate financial behavior to a hot topic. Existing research mainly explores the impact of the following three types of managers’ characteristics on social responsibility. First one is demographic background, which mainly focuses on examining the impact of personal characteristics on social responsibility [10] such as age, gender, education, tenure, social responsibility and so on [10]. Second is psychological characteristics, such as managers’ overconfidence [11], managers’ narcissism [12] and so on. Third is the acquired experience, such as overseas experience [3], and poverty experience [4].

All these individual characteristics are used to portray the differentiation and diversification of the cognitive structure and value concept of the heterogeneous and bounded rational managers, and culture is a deep-seated factor in the formation of managers’ cognitive structure and values. China provides an excellent research context for the research on this topic. Confucian culture is the mainstream culture of the Chinese nation with five-thousand-year civilization, and its thinking has always been the most basic mainstream value of the Chinese nation. The values advocating ‘propriety, righteousness, integrity, shame, benevolence, love, loyalty, and filial piety’ are still the norms for most Chinese people to act. The Confucian culture has also profoundly affected the behavior of Chinese companies. Studies have shown that Confucian culture reduces the level of risk-taking [13], improves the quality of internal control [14] and the level of social responsibility information disclosure [15]. In addition, the Communist culture, which has grown rapidly and has a strong vitality in the past century, has also attracted the attention of the academic circles. Existing research shows that private companies will assume more social responsibilities such as investing more resources in pollution control and environmental protection [16,17], or more charitable donations [18] by building grassroots Party organizations or encouraging senior executives to join the CPC accepting the influence of Communist culture.

Targeted poverty alleviation is a special social responsibility given to Chinese companies in this era. Only a few scholars have paid attention to the impact of individual characteristics of managers on targeted poverty alleviation. The particularity of targeted poverty alleviation is that for state-owned companies, it is a political task, unlike private company, and it is more of a voluntary act. Whether the identity of a chairman’s CPC membership of a private company will enable him to recognize the values of Communism and take the initiative to assume the responsibility of targeted poverty alleviation is a question that needs to be tested empirically. Therefore, based on the perspective of Communist culture, this paper studies the influence of private executives’ identity of the CPC membership on targeted poverty alleviation, to provide a useful supplement to the research in this field.

#### 2.2.1. The influence of the chairman’s identity of the CPC membership of a private company on targeted poverty alleviation

The political beliefs of business managers or founders will have an important impact on business decision-making [19]. The empirical evidence from the U.S. market shows that representatives of different parties have different attitudes towards risk, there are significant differences in business strategies [20], tax avoidance behaviors [21,22], mergers and acquisitions [23], and other aspects of companies managed or created by supporters of the Democratic and Republican parties. Different from the capitalist system of Western countries, China is a socialist country under the leadership of the CPC. The dominance of the public economy is the main feature of the socialist economy with Chinese characteristics, and the private economy is an important part. State-owned companies representing the public economy not only have a sound governance mechanism for the Party organizations, but most of their executives are held by the CPC members with strong political qualities; while for private companies representing the private economy, the construction of grassroots Party organizations is voluntary. There is also no mandatory requirement for Party membership for senior management positions. Given this, the research of political culture and corporate behavior based on the Chinese background also discusses state-owned companies and private companies separately. For state-owned companies, existing research mainly measures the degree of overlap (two-way entry, cross-service) between primary Party organizations and corporate governance organizations (board of directors, board of supervisors, and management) to measure Party organizations’ participation in corporate governance. The research found that it will have an important impact on executive compensation contracts [24] and executive corruption [25]. For private companies, the existing research mainly observes the political participation of private companies indirectly from political organization building or executives’ identity of the CPC membership, and studies have found that it has an important impact on charitable donations [8], financial violations [26], social responsibility [8] and other aspects.

Communist culture is the macro-political and cultural environment of the socialist economy with Chinese characteristics. This also determines that no matter how China’s economy is reformed and developed, the principle that the public economy dominates will never change. Besides, the CPC members are the carrier of this cultural inheritance, and the identity of the CPC members of the executives will subtly influence their cognitions, interactions and decision-making [4], and have an important impact on their business decision-making. This paper reveals that private companies managed by the CPC members will more actively assume the responsibility of targeted poverty alleviation and invest more funds for it. The reasons are as follows.

Firstly, the identity of the CPC membership makes the chairman agree with the values of Communism more. From an individual’s application for CPC member, a strict and lengthy process of review and training is required. All party members and comrades are people with lofty ideals who support the leadership of the CPC and have lofty Communist beliefs. In addition, the CPC members should participate in the Party’s organizational life and receive education from the organization. This is an important system guarantee for Party members to maintain their advanced nature. Existing research shows that in China, entrepreneurs with a background as the CPC member possess Communist values [27,28], and Communist culture itself has the spirit of altruism and dedication to ‘serve the people wholeheartedly’, and this value concept will be internalized In the thinking mode of Party members and entrepreneurs, which positively affects their business decisions. Existing studies have also found that it is the entrepreneurs who are members of CPC to undertake more social responsibilities. Targeted poverty alleviation is a national strategy put forward in response to China’s poverty alleviation process at the critical stage, and it directly points to eliminating polarization and achieving common prosperity, which is the essential requirement of Socialism. Therefore, the identity of a chairman’s CPC membership of private company will enable them to endorse this strategy more willingly, and actively participate in targeted poverty alleviation, give play to its own advantages, and assume this special social responsibility.

Secondly, the chairman who is a CPC member will pay more attention to the reputation of himself and the company. Communist culture also inherently requires CPC members to have an awareness of excellent ‘vanguard model’ and higher moral standards [7], play a leading role in all aspects, and reflect the advanced nature of Party members. Specific to the targeted poverty alleviation that this article focuses on, to ensure a full victory in the targeted poverty alleviation in 2020, under the active call of the government, a large-scale poverty alleviation pattern with the active participation of all sectors of society has gradually formed. Chinese government put forward a series of policies to disclose the information of targeted poverty alleviation in the social responsibility section of their annual reports. It helps guarantee listed companies’ indispensable role in fulfilling their duties for targeted poverty alleviation. Under such background, the targeted poverty alleviation has become the focus of attention from all walks of life. The chairman of the CPC members of private companies who have always been a ‘pioneer model’ also bears more social attention and higher expectations. Therefore, regardless of individual or corporate reputation, the chairman of the CPC membership of private companies should be more actively involved in targeted poverty alleviation in order to maintain a consistently good social image.

Based on the above analysis, this paper proposes the following research hypotheses:

*Hypothesis 1*: Compared with other private companies, private companies in which chairman is a CPC member will be more actively involved in targeted poverty alleviation and invest more in poverty alleviation funds.

#### 2.2.2. Chairman’s CPC membership, Party organization building, and targeted poverty alleviation by private companies

The Party organization is an important channel through which the chairman’s CPC membership of a private company plays a role in promoting targeted poverty alleviation. Firstly, the construction of Party organizations in private companies is a concrete manifestation of the mutual embedding of political organizations and economic organizations [29], by setting up grassroots party organizations to strengthen the construction of corporate political culture and further reinforce the role of the chairman’s CPC membership of private companies in promoting targeted poverty alleviation. Secondly, the establishment of political organizations facilitates the establishment of ties between private companies and the government, and provides opportunities for private companies to engage in the discussion of politics. In the meantime, the Party organization of private company is also a window for private companies to connect with the government’s national policies and guidelines. It can better convey the policies and guidelines of the Party and the government, thus private companies can accept and actively respond to the relevant government departments’ initiatives on targeted poverty alleviation, which forms a synergy with the chairman’s CPC membership to better fulfill the responsibility of targeted poverty alleviation. We previously conducted a special survey on targeted poverty alleviation of more than 20 listed companies and found a very interesting phenomenon. For private companies that have set up grassroots Party organizations, the grassroots Party branches are responsible for their targeted poverty alleviation work, while the general trade unions are responsible for private companies that have not set up grassroots Party organizations. Third, targeted poverty alleviation is not only a special social responsibility, but also a political task that must be completed for Party and government cadres at all levels. The construction of grassroots Party organizations is also convenient for the chairman of private companies to accept and complete this political task. Based on the above analysis, this article proposes the following research hypotheses:

*Hypothesis 2*: Compared with private companies that have not set up grassroots Party organizations, in private companies that have set up grassroots Party organizations, the chairman’s CPC member status will promote targeted poverty alleviation even more significantly.

## 3. Empirical research

### 3.1. Sample selection and data source

This paper selects the A-share private listed companies in Shanghai and Shenzhen Stock Exchanges from 2016 to 2020 as the initial sample. In September 2016, China’s Securities Regulatory Commission encouraged the capital market and listed companies to participate in precise poverty alleviation. Aiming to help the poor precisely, in December 2016, the Shanghai and Shenzhen stock markets in China simultaneously issued a notice requiring listed companies to disclose information on precise poverty alleviation in the social responsibility section of their annual reports. Moreover, a comprehensive victory in the fight against poverty and the complete elimination of absolute poverty has made in 2020. Therefore, we choose the period of sample from 2016 to 2020.

We exclude the companies of financial and insurance industries, ST and *ST, and companies that lack financial information. As a result, a total of 9,889 valid samples collected in 5 years were obtained. The identity data of the CPC members of the board of directors of private companies required for this study is non-mandatory disclosure information. We mainly obtain it manually through channels such as the WIND database, corporate website, Baidu Encyclopedia, Sina Finance, and Juchao Information. Targeted poverty alleviation data comes from the CNRDS database; other variables are all from the CSMAR database. Moreover, all continuous variables are winsorized at the 1% and 99% quantiles to alleviate the influence of extreme values on the results.

### 3.2. The variables

#### 3.2.1. The dependent variables

1. *PA_D*
  Whether to participate in the precision poverty alleviation dummy variable (*PA_D*), if it participates in the targeted poverty alleviation in the current year, the value is 1, otherwise it is 0.
2. *PA_A*

The investment amount of targeted poverty alleviation (*PA_A*), equals to the natural logarithm of the total amount invested in the current year.

#### 3.2.2. The independent variables

1. *CCPC*
  Taking the leaders of the decision-making level, the chairman of the board as the object of investigation, constructing the dummy variable *CCPC* as chairman’s CPC member status. If the chairman is a member of the CPC, the value is 1; otherwise, is taken the value as 0.
2. *Party*

Party organization building (*Party*) reflects the information whether a private listed company has established a grassroots CPC organization. If a grassroots CPC organization has been set up in the year, the value is 1. Otherwise, its value is 0.

#### 3.2.3. Controlled variable

The control variables mainly include the following three categories. (1) The control variables related to the individual characteristics of the chairman of the board [30], including: age (*Age*), gender (*Gender*), education (*Edu*), shareholding ratio (*Share*), and two-job integration (*Dual*). (2) Firm-level control variables [31,32], including firm size (*Size*), asset-liability ratio (*Lev*), profitability (*RoA*), operating capability (*OC*), corporate growth (*Growth*), holdings of the largest shareholder Share ratio (*First*), independent director ratio (*Inde*) and listing age (*FirmAge*). (3) Annual and industry control variables. The main reason of poverty lies in the huge gap between rich and poor caused by uneven regional economic development should be considered. In addition, from the perspective of listed companies participation in targeted poverty alleviation, there is serious regional mismatch between poverty alleviation resources and poverty alleviation needs, poverty alleviation may vary greatly between different regions. Therefore, regional control variables are also added to the model. Specific variable definitions are shown in Table 1.

**Table 1:** The definition of variables.

Type of variable	symbol	Variable name and metric
Dependent variable	*PA_D*	Whether to participate in targeted poverty alleviation, private companies participating in targeted poverty alleviation is 1, otherwise it is 0
	*PA_A*	The amount of targeted poverty alleviation investment is the natural logarithm of the total amount of private companies targeted poverty alleviation investment (Ten thousand RMB)
Independent variable	*CCPC*	Chairman Communist Party of China membership, if chairman of private companies is a CPC member, the value is 1, otherwise it is 0
	*Party*	Party organization construction, private companies in that year set up grassroots CPC organizations will take the value of 1, otherwise it is 0
Controlled variable	*Age*	Age, the natural log of the age of the chairman
	*Gender*	Gender, with a value of 1 when the chairman was male and 0 for female
	*Edu*	Education, 1 is for technical secondary school and below, 2 is for junior college, 3 is for bachelor’s degree, 4 is graduate student
	*Share*	Holding proportion, the number of shares held by the chairman divided by the total number of shares
	*Dual*	The two positions are in one, and the chairman and CEO positions are worth 1, otherwise it is 0
	*Size*	Firm size, the natural log of the total assets of the company
	*Lev*	Financial leverage, liabilities divided by total assets
	*Roa*	Profitability, net profit divided by total assets
	*OC*	Asset operating capacity, operating revenue divided by the average total assets
	*Growth*	Firm growth, the growth rate of operating revenue
	*First*	The shareholding proportion of the largest shareholder, the shareholding percentage of the largest shareholder
	*Inde*	The proportion of independent directors, the proportion of independent directors of the board of directors
	*FirmAge*	The age of listing, the year since the listing time of private firms
	*Year*	Annual variable, taking a value of 1 when the observation is a given year, otherwise 0
	*Ind*	Industry variable, value 1 when the observation belongs to a particular industry, or otherwise 0
	*Area*	Regional variable, taking a value of 1 when the observation value is registered to a particular province and city, or 0 otherwise

### 3.3. Model

In order to test the above-mentioned two research hypotheses, this paper constructs the following multiple regression model:

$$\mathrm{PA}=\alpha_{0}+\alpha_{1}\mathrm{CCPC}+\sum \alpha_{i}\mathrm{Controls}+\varepsilon$$ (1)

Controls stand for control variables. The dependent variables include two of *PA_D* and *PA_A*. *PA_D* is a dummy variable, the Logit regression model is used. *PA_A* gathers a large number of samples at 0 and Tobit regression model is used. When verifying research hypothesis 1, the coefficient α_1_ of *CCPC* is expected to be significantly positive. To verify research hypothesis 2, it is expected that the coefficient of *CCPC* is more significant in private companies that have set up grassroots organizations of CPC, according to the Party organization construction (*Party*) variable group regression.

## 4. Empirical results

### 4.1. Descriptive statistics

Table 2 shows the descriptive statistics of the variables including 9,889 observations. The mean value of *PA_D* is 0.183, suggesting that 18.3% of the observed private companies participate in targeted poverty alleviation and there is still a lot of room to explore. Moreover, it also shows that private companies are important force for targeted poverty alleviation. The standard deviation of *PA_A* is 5.342, indicating that the average investment in poverty alleviation funds of private companies participating in targeted poverty alleviation with large differences. The mean of *CCPC* of private companies is 31.1%, reflecting that although there is no requirement on whether the chairman of a private company is a member of the CPC, more than a quarter of the private corporate chairmen are still the CPC members. Part of this is the formation of the original government officials doing business and the reform of state-owned corporates, and some of them are private entrepreneurs who joined the CPC in the later period. Other control variables are consistent with previous studies.

**Table 2:** Descriptive statistical results.

Panel A: Descriptive statistical results of the whole sample								
**Variables**	**N**	**Mean**	**Std**	**Min**	**Quantile 25**	**Median**	**Quantile 75**	**Max**
*PA_D*	9889	0.183	0.387	0	0	0	0	1
*PA_A*	9889	2.491	5.342	0	0	0	0	17.623
*CCPC*	9889	0.311	0.463	0	0	0	1	1
*Party*	9889	0.693	0.461	0	0	1	1	1
*Age*	9889	3.986	0.156	3.219	3.912	4.007	4.078	4.443
*Gender*	9889	0.935	0.247	0	1	1	1	1
*Edu*	9889	3.415	0.844	0	3	4	4	4
*Share*	9889	0.129	0.152	0	0	0.06	0.23	0.56
*Dual*	9889	0.378	0.485	0	0	0	1	1
*Size*	9889	22.005	1.092	19.735	21.225	21.905	22.661	25.342
*Lev*	9889	0.399	0.199	0.06	0.239	0.385	0.53	0.962
*Roa*	9889	0.03	0.097	-0.532	0.015	0.04	0.07	0.211
*OC*	9889	0.608	0.389	0.058	0.355	0.529	0.76	2.354
*Growth*	9889	0.189	0.478	-0.682	-0.023	0.116	0.29	3.099
*First*	9889	0.306	0.131	0.081	0.205	0.291	0.388	0.671
*Inde*	9889	0.379	0.052	0.333	0.333	0.364	0.429	0.571
*FirmAge*	9889	2.891	0.32	0.693	2.708	2.944	3.135	4.143

Panel B: The univariate test results			
**Variable**	**Sample of the chairman of the CPC members**	**Sample of non-CPC member chairman**	**T-test (T-Value)**
*PA_D*	0.240	0.157	0.082*** (9.832)
*PA_A*	3.306	2.123	1.182*** (10.252)
Sample number	3075	6814	—

Note: *, * *, * * * indicate the significance levels of 10%, 5%, and 1%, respectively.

Panel B shows the test result of the univariate of targeted poverty alleviation between the sample of a CPC member and non-CPC chairman. Compared with the non-CPC chairman, if the chairman of the board is a member of the CPC, the willingness to participate and the amount of investment in targeted poverty alleviation of private companies are significantly greater, which implies that the chairman’s CPC membership of private companies plays a role in promoting targeted poverty alleviation.

### 4.2. Correlation coefficient analysis

As the data shown in Table 3, the correlation coefficient between *CCPC* and *PA_D* is 0.098, which is significant at the 1% level. It implies that the chairman’s CPC membership is significantly positively correlated with the willingness of private companies to participate in targeted poverty alleviation. Moreover, the correlation coefficient between *CCPC* and *PA_A* is 0.103, which is significant at the 1% level. It implies that the chairman’s CPC membership is significantly positively correlated with the investment in targeted poverty alleviation in private companies. The above results support hypothesis 1. In addition, the correlation coefficients between all the variables are not above 0.5 except between *PA_D* and *PA_A*, suggesting that the subsequent regression conclusions are less affected by multicollinearity.

**Table 3:** Results of the correlation coefficient analysis.

Variable	*PA_D*	*PA_A*	*CCPC*	*Party*	*Age*	*Gender*	*Edu*	*Share*	*Dual*	*Size*	*Lev*	*Roa*	*OC*	*Growth*	*First*	*Inde*	*FirmAge*
*PA_D*	1																
*PA_A*	0.985***	1															
*CCPC*	0.098***	0.103***	1														
*Party*	0.098***	0.104***	0.234***														
*Age*	0.030***	0.032***	0.120***	0.043***	1												
*Gender*	-0.025**	-0.021**	0.024**	0.044***	0.077***	1											
*Edu*	0.037***	0.040***	-0.014	-0.002	-0.188***	-0.0.005	1										
*Share*	-0.070***	-0.076***	-0.102***	-0.060***	0.094***	0.041***	-0.113***	1									
*Dual*	-0.030***	-0.032***	-0.097***	-0.051***	-0.118***	0.014	0.030***	0.200***	1								
*Size*	0.188***	0.222***	0.120***	0.145***	0.034***	0.013	0.082***	-0.228***	-0.111***	1							
*Lev*	0.053***	0.066***	0.052***	0.066***	-0.096***	0.021**	0.095***	-0.185***	-0.076***	0.432***	1						
*Roa*	0.074***	0.078***	0.023**	0.046***	0.086***	-0.010	-0.054***	0.136***	0.021**	0.058***	-0.350***	1					
*OC*	0.036***	0.038***	0.006	0.042***	-0.004	0.013	-0.002	0.023**	-0.003	0.064***	0.098***	0.210***	1				
*Growth*	-0.013	-0.012	-0.014	-0.017*-	-0.048***	0.010	0.023	0.036***	0.015	0.075***	0.026**	0.236***	0.197***	1			
*First*	0.021**	0.027***	0.028***	0.031***	0.034***	-0.047***	-0.075***	0.195***	0.068***	0.043***	-0.016	0.167***	0.117***	0.017*	1		
*Inde*	-0.029***	-0.031**	-0.064***	-0.064***	-0.047***	-0.041***	-0.035**	0.103***	0.136***	-0.089***	-0.012	-0.031***	-0.008	-0.004	0.039***	1	
*FirmAge*	0.083***	0.081***	0.108***	0.063***	0.048***	-0.038***	0.085***	-0.181***	-0.088***	0.119***	0.127***	-0.069***	-0.035***	-0.052***	-0.057***	-0.044***	1

Note: *, * *, * * * indicate the significance levels of 10%, 5%, and 1%, respectively.

### 4.3. Regression results

Table 4 illustrates the regression results for Model (1). Column (2) introduces control variables based on column (1), while Column (4) introduces control variables based on column (3) to make model more accurate. For regression of the four CCPC, the coefficients are all significant. Moreover, in column (2), the coefficient of CCPC is 0.3432, and it is significant at the 1% level, which shows that the chairman’s CPC membership can significantly increase the willingness of private companies to participate in targeted poverty alleviation. In column (4), the coefficient of *CCPC* is 0.7206, which is also significant at the 1% level. This shows that the chairman’s CPC members can also increase the amount of investment in targeted poverty alleviation in private companies. The above conclusions support research hypothesis 1.

**Table 4:** The regression results of the influence of chairman’s CPC membership on targeted poverty alleviation in private companies.

Variable	*PA_D*		*PA_A*	
	(1)	(2)	(3)	(4)
*CCPC*	0.4624***	0.3432***	1.0162***	0.7206***
	(8.3588)	(5.9375)	(8.8452)	(6.3086)
*Age*		0.2299		0.3727
		(1.1926)		(1.0694)
*Gender*		-0.2718***		-0.4972**
		(-2.5769)		(-2.3673)
*Edu*		0.0753**		0.1482**
		(2.1626)		(2.3399)
*Share*		-0.4805**		-0.9336**
		(-2.3266)		(-2.4822)
*Dual*		0.0581		0.1033
		(0.9611)		(0.9315)
*Size*		0.3927***		0.9834***
		(13.1954)		(17.4814)
*Lev*		-0.0038		-0.1204
		(-0.0206)		(-0.3551)
*Roa*		2.7199***		3.6742***
		(5.6214)		(5.7876)
*OC*		0.0799		0.2109
		(0.9952)		(1.3850)
*Growth*		-0.1657***		-0.3267***
		(-2.6176)		(-2.8299)
*First*		-0.0223		0.3251
		(-0.1011)		(0.7768)
*Inde*		-0.3691		-0.4791
		(-0.6774)		(-0.4760)
*FirmAge*		0.2936***		0.4793***
		(2.9868)		(2.7967)
*Constant*	-1.7315***	-12.1239***	2.7049***	-21.8640***
	(-6.9009)	(-11.4323)	(5.0981)	(-11.1287)
*Year*	control	control	control	control
*Industry*	control	control	control	control
*Area*	control	control	control	control
*Observations*	9889	9889	9889	9889
*Pseudo R2*	0.0419	0.0807	0.0073	0.0304
*Wald Chi2/LR Chi2*	381.43	644.46	446.33	949.15

Note: *, * *, * * * indicate the significance levels of 10%, 5%, and 1%, respectively. When the explained variable is *PA_D*, using the Logit model, the Z value of the coefficient is reported under the coefficient. When the explained variable is *PA_A*, the Tobit model is used, and the T value is reported in the square bracket under the coefficient (the same below).

From control variables of individual characteristics of the chairman, it takes column (2) as an example. The results show that female chairmen with higher education can promote the targeted poverty alleviation of private companies, while the chairman’s shareholding ratio inhibits private companies fulfill their responsibilities for targeted poverty alleviation, similar with column (4). From the perspective of firm-level control variables, private companies with larger scale, stronger profitability, better growth and firm ages will more actively participate in targeted poverty alleviation with greater poverty alleviation efforts.

## 5. Mechanism: Party organizations

Table 5 shows the regression results of the influence of chairman’s CPC membership on targeted poverty alleviation in private companies whether having been set up Party organizations. First, based on the method of group regression, the results are shown in column (2), (3), (5) and (6). Second, to make the conclusion more valid, we put the dummy variables for the Party branch setup in the model and moderate the impact of the CPC status. The results are presented in column (1) and (4). According to Table 5, in column (2), the coefficient of *CCPC* is 0.3259, which is significant at the 1% level. In column (3), the coefficient of *CCPC* is not significant. This indicates that in private companies with Party organizations, the status of chairman’s CPC can significantly increase the willingness of companies to participate in targeted poverty alleviation. In private companies without a Party organization, the chairman’s CPC membership reduces the willingness of the company to participate in targeted poverty alleviation. In column (5), the coefficient of *CCPC* is 0.7239 and is significant at the 1% level while in column (6), the coefficient of *CCPC* is not significant. It shows that in private companies with the Party organizations, the chairman’s CPC members will have a significant positive effect on the company’s investment in targeted poverty alleviation. Otherwise, it will have a significant negative effect. Furthermore, by investigating the coefficient of interactive term between *CCPC* and the above dummy, it finds out that in column (1) the coefficient of CCPC*Party is 0.2686, significant at the 10% level, and in column (4) the coefficient of CCPC*Party is 0.7819, significant at the 1% level, which support the above conclusions.

**Table 5:** The channel role of CPC organization building.

Variable	*PA_D*			*PA_A*		
	Full sample	Party = 1	Party = 0	Full sample	Party = 1	Party = 0
	(1)	(2)	(3)	(4)	(5)	(6)
*CCPC*	0.0518	0.3259***	-0.0653	-0.0306	0.7239***	-0.0995
	(0.3497)	(5.0571)	(-0.4167)	(-0.1166)	(5.2521)	(-0.4433)
CCPC*Party	0.2686*			0.7819***		
	(1.6809)			(2.6925)		
*Party*	0.2793***			0.4060***		
	(3.6949)			(3.1455)		
*Age*	0.2385	0.0304	0.8094*	0.3886	0.0194	1.0619**
	(1.2382)	(0.1363)	(1.9465)	(1.1167)	(0.0439)	(2.0074)
*Gender*	-0.2921***	-0.1860	-0.5326***	-0.5442***	-0.4521	-0.6621**
	(-2.7411)	(-1.4285)	(-2.7837)	(-2.5926)	(-1.5991)	(-2.3142)
*Edu*	0.0753**	0.1138***	-0.0244	0.1486**	0.2260***	-0.0015
	(2.1584)	(2.7986)	(-0.3513)	(2.3492)	(2.8280)	(-0.0151)
*Share*	-0.4738**	-0.4412*	-0.5757	-0.9163**	-0.8958*	-1.0001*
	(-2.2848)	(-1.8309)	(-1.3611)	(-2.4391)	(-1.8431)	(-1.8047)
*Dual*	0.0587	0.1516**	-0.2884**	0.1030	0.3109**	-0.3503**
	(0.9686)	(2.2057)	(-2.1555)	(0.9306)	(2.1984)	(-2.0991)
*Size*	0.3830***	0.4040***	0.3441***	0.9615***	1.0989***	0.6532***
	(12.8350)	(11.6525)	(5.6038)	(17.0485)	(15.3551)	(7.5833)
*Lev*	-0.0159	-0.2074	0.6102	-0.1495	-0.4716	0.7261
	(-0.0868)	(-0.9691)	(1.6425)	(-0.4414)	(-1.0669)	(1.4744)
*Roa*	2.6349***	2.1139***	4.2301***	3.5222***	3.5612***	3.7200***
	(5.4644)	(4.1712)	(3.3270)	(5.5514)	(4.1686)	(4.3101)
*OC*	0.0722	0.0700	-0.0278	0.1958	0.2251	-0.0178
	(0.8980)	(0.7725)	(-0.1558)	(1.2877)	(1.1630)	(-0.0768)
*Growth*	-0.1610**	-0.1499**	-0.1961	-0.3208***	-0.3419**	-0.3190**
	(-2.5284)	(-2.0170)	(-1.5902)	(-2.7814)	(-2.1995)	(-2.0392)
*First*	-0.0353	-0.0831	0.4050	0.3216	0.1735	0.7983
	(-0.1601)	(-0.3391)	(0.7876)	(0.7692)	(0.3341)	(1.1777)
*Inde*	-0.2668	-0.3288	0.0478	-0.3142	-0.2941	-0.0900
	(-0.4887)	(-0.5088)	(0.0441)	(-0.3124)	(-0.2247)	(-0.0619)
*FirmAge*	0.2745***	0.4237***	-0.3837*	0.4508***	0.7408***	-0.5646**
	(2.8157)	(3.6360)	(-1.9335)	(2.6327)	(3.4416)	(-2.0998)
*_cons*	-12.0756***	-12.0395***	-11.1544***	-21.6257***	-23.8865***	-14.1712***
	(-11.4215)	(-9.8760)	(-4.9634)	(-11.0212)	(-9.6785)	(-4.6098)
*Year*	Control	Control	Control	Control	Control	Control
*Industry*	Control	Control	Control	Control	Control	Control
*Area*	Control	Control	Control	Control	Control	Control
*Observations*	9889	6855	3034	9889	6855	3034
*Pseudo R2*	0.0839	0.0803	0.0954	0.0160	0.0162	0.0157
*Wald Chi2/LR Chi2*	695.67	511.31	192.87	979.58	701.89	276.29

Combining the results of column (1) to (6), it implies that the grassroots Party branch is an important channel to play the role of the chairman’s CPC membership to promote targeted poverty alleviation in private companies. For private companies, having established grassroots Party organizations in these companies, the individual recognition of the Communist cultural values and the importance of the self-reputation, will be reflected in the targeted poverty alleviation responsibility. At this moment, the chairman’s CPC identity can speak out through Party organizations, and the Party organization is specifically responsible for the implementation of targeted poverty alleviation decisions. Moreover, it is also possible that after the establishment of grassroots Party branches, the governance mechanism of private companies is closer to that of state-owned companies, through the cross-employment of the Party organization and the corporate governance organization to strengthen the influence of the chairman’s CPC membership on the performance of targeted poverty alleviation responsibilities in private companies. On the contrary, the chairman’s CPC membership has even a negative effect on targeted poverty alleviation. Therefore, research hypothesis 2 has been supported.

## 6. Robustness test and endogenous problems

### 6.1. Replace dependent variables

Table 6 reveals the robustness test results of adjusting the sample range. In previous research, we used the natural logarithm of the amount of investment in targeted poverty alleviation by private companies to describe the level of investment in targeted poverty alleviation to describe its relevant level. It is an absolute quantitative indicator, with insufficient comparability between companies of different sizes, which may have a certain impact on the previous research conclusions. Through the regression results of the previous article, the regression results of the firm-scale control variables also show that the firm-scale is an important factor that affects the degree of investment in targeted poverty alleviation by private companies. Therefore, in order to verify the robustness of the research conclusions of this paper, the previous research conclusions were re-examined after standardizing the investment amount of precision poverty alleviation with total assets. Thus, it can be found that after replacing the dependent variable, all the research conclusions remain robust.

**Table 6:** The robustness test results of replacing independent variables.

Variable	*PA_A*			
	Full sample	Full sample	Party = 1	Party = 0
	(1)	(2)	(3)	(4)
*CCPC*	0.0023**	-0.0032**	0.0023*	-0.0040**
	(2.1557)	(-2.2212)	(1.8244)	(-2.5451)
CCPC*Party		0.0056***		
		(2.9365)		
*Party*		0.0034***		
		(3.2994)		
*Age*	0.0065**	0.0067**	0.0036	0.0136***
	(2.0899)	(2.1292)	(0.8809)	(3.3464)
*Gender*	-0.0009	-0.0012	-0.0002	-0.0025
	(-0.4286)	(-0.6154)	(-0.0605)	(-0.9981)
*Edu*	0.0017***	0.0017***	0.0020***	0.0010*
	(3.3277)	(3.3393)	(2.9391)	(1.7438)
*Share*	-0.0017	-0.0015	-0.0005	-0.0047
	(-0.4935)	(-0.4477)	(-0.1078)	(-1.0770)
*Dual*	0.0003	0.0003	0.0015	-0.0028**
	(0.2848)	(0.2892)	(1.1661)	(-2.2458)
*Size*	0.0029***	0.0027***	0.0031***	0.0021***
	(4.7528)	(4.4412)	(3.7888)	(2.9527)
*Lev*	0.0025	0.0022	0.0009	0.0071**
	(0.8648)	(0.7837)	(0.2192)	(2.2799)
*Roa*	0.0206***	0.0194***	0.0243***	0.0119**
	(5.2460)	(4.9551)	(4.4126)	(2.5198)
*OC*	0.0040**	0.0039**	0.0063***	-0.0027
	(2.5130)	(2.4440)	(2.9747)	(-1.5550)
*Growth*	-0.0031***	-0.0030***	-0.0042***	-0.0011
	(-4.5921)	(-4.5164)	(-4.2689)	(-1.6117)
*First*	0.0016	0.0016	-0.0003	0.0072
	(0.3949)	(0.3806)	(-0.0618)	(1.0784)
*Inde*	-0.0066	-0.0053	-0.0028	0.0022
	(-0.8003)	(-0.6387)	(-0.2393)	(0.2548)
*FirmAge*	-0.0008	-0.0011	-0.0002	-0.0040**
	(-0.4958)	(-0.6268)	(-0.0876)	(-2.1183)
*_cons*	-0.0619***	-0.0600***	-0.0522**	-0.0812***
	(-3.0977)	(-3.0004)	(-1.9850)	(-3.4136)
*Year*	Control	Control	Control	Control
*Industry*	Control	Control	Control	Control
*Area*	Control	Control	Control	Control
*Observations*	9889	9889	6855	3034
*R-squared*	0.038	0.040	0.047	0.017
*F-Value*	8.97	8.44	7.58	2.57

### 6.2. Adjusting the sample range

Table 7 represents the robustness test results of adjusting the sample range. The duration from 2016 to 2020 includes the outbreak of Covid-19 since the end of 2019. Covid-19 caused a big impact on companies all over the world, and many of them were in business difficulties including Chinese companies. In order to fight against the epidemic, companies were likely to abnormally reduce target poverty alleviation in 2020, thereby creating a braking effect. The braking effect might make the results unreliable. So, we deleted the data of year 2020 from the samples and conducted empirical regression based on data from 2016 to 2019. The results are shown in Table 7. The coefficients of *CCPC* in column (1) and (2) are positive, significant at the 1% level. The coefficients of CCPC*Party and *party* in column (3) and (6) are positive and significant. The column (4) and (5), (7) and (8) are consistent with the former regression model in groups. Therefore, the conclusions are still valid.

**Table 7:** The robustness test results of adjusting the sample range.

Variable	*PA_D*	*PA_A*	*PA_D*			*PA_A*		
	Full sample	Full sample	Full sample	Party = 1	Party = 0	Full sample	Party = 1	Party = 0
	(1)	(2)	(3)	(4)	(5)	(6)	(7)	(8)
*CCPC*	0.3274***	0.6511***	-0.0051	0.3100***	-0.1264	-0.1210	0.6454***	-0.1826
	(4.9313)	(5.1078)	(-0.0299)	(4.1656)	(-0.7099)	(-0.4191)	(4.1609)	(-0.7399)
CCPC*Party			0.3098*			0.7988**		
			(1.6858)			(2.4903)		
*Party*			0.2877***			0.4303***		
			(3.3282)			(2.9913)		
*Age*	0.3868*	0.6567*	0.3871*	0.2267	0.7295	0.6603*	0.4072	1.0216*
	(1.7095)	(1.6673)	(1.7101)	(0.8565)	(1.5630)	(1.6793)	(0.8044)	(1.7559)
*Gender*	-0.3078**	-0.5658**	-0.3265***	-0.2569*	-0.4753**	-0.6136***	-0.6236*	-0.5407*
	(-2.5400)	(-2.3816)	(-2.6659)	(-1.7213)	(-2.1481)	(-2.5850)	(-1.9400)	(-1.6807)
*Edu*	0.0693*	0.1351**	0.0690*	0.1099**	-0.0494	0.1357**	0.2155**	-0.0322
	(1.7827)	(1.9604)	(1.7733)	(2.4130)	(-0.6396)	(1.9725)	(2.4715)	(-0.3046)
*Share*	-0.5491**	-0.9962**	-0.5406**	-0.3867	-1.0023**	-0.9755**	-0.7183	-1.4254**
	(-2.3193)	(-2.3826)	(-2.2763)	(-1.4011)	(-2.0517)	(-2.3367)	(-1.3234)	(-2.3269)
*Dual*	0.0694	0.1010	0.0695	0.1527*	-0.2340	0.1009	0.2891*	-0.2858
	(0.9916)	(0.8118)	(0.9936)	(1.9087)	(-1.5347)	(0.8122)	(1.8085)	(-1.5420)
*Size*	0.3745***	0.9062***	0.3649***	0.3913***	0.3051***	0.8856***	1.0325***	0.5584***
	(10.6509)	(14.1846)	(10.3628)	(9.5391)	(4.2630)	(13.8399)	(12.5895)	(5.8240)
*Lev*	-0.0515	-0.1514	-0.0580	-0.1938	0.4811	-0.1748	-0.3703	0.6352
	(-0.2398)	(-0.3952)	(-0.2706)	(-0.7665)	(1.1480)	(-0.4572)	(-0.7357)	(1.1558)
*Roa*	2.5189***	3.4445***	2.4387***	2.2906***	3.0212**	3.2919***	3.7991***	3.0481***
	(4.3607)	(4.8062)	(4.2397)	(3.6359)	(2.4379)	(4.5972)	(3.8999)	(3.1657)
*OC*	0.0837	0.2002	0.0739	0.0583	-0.0074	0.1809	0.1975	-0.0279
	(0.9169)	(1.1852)	(0.8082)	(0.5641)	(-0.0368)	(1.0721)	(0.9152)	(-0.1092)
*Growth*	-0.1899**	-0.3628***	-0.1868**	-0.1801**	-0.2151	-0.3591***	-0.3868**	-0.3522**
	(-2.5386)	(-2.8902)	(-2.4823)	(-2.0508)	(-1.5165)	(-2.8644)	(-2.2710)	(-2.0879)
*First*	0.0227	0.4058	0.0037	-0.0231	0.3543	0.3915	0.2534	0.7928
	(0.0891)	(0.8688)	(0.0147)	(-0.0815)	(0.6055)	(0.8390)	(0.4347)	(1.0608)
*Inde*	-0.3809	-0.5029	-0.2725	-0.5012	0.5455	-0.3248	-0.4665	0.1962
	(-0.5983)	(-0.4463)	(-0.4267)	(-0.6573)	(0.4449)	(-0.2886)	(-0.3143)	(0.1230)
*FirmAge*	0.2708**	0.4231**	0.2530**	0.3842***	-0.3634	0.3993**	0.6468***	-0.5171*
	(2.4231)	(2.2449)	(2.2846)	(2.8970)	(-1.6073)	(2.1213)	(2.7168)	(-1.7682)
*_cons*	-12.2482***	-21.1447***	-12.1816***	-12.4085***	-10.1071***	-20.9201***	-23.7400***	-12.1085***
	(-9.7627)	(-9.4876)	(-9.7379)	(-8.5334)	(-3.9042)	(-9.4010)	(-8.3927)	(-3.5466)
*Year*	Control	Control	Control	Control	Control	Control	Control	Control
*Industry*	Control	Control	Control	Control	Control	Control	Control	Control
*Area*	Control	Control	Control	Control	Control	Control	Control	Control
*Observations*	7680	7680	7680	5255	2425	7680	5255	2425
*Pseudo R2*	0.0799	0.0149	0.0834	0.0822	0.0900	0.0155	0.0162	0.0145
*Wald Chi2/LR Chi2*	516.42	705.84	535.76	400.36	147.45	732.97	533.75	202.67

### 6.3. PSM-paired samples

Table 8 shows the results of the PSM robustness test. In order to alleviate the impact of the endogenous problems caused by self-selection on the conclusions of this research, we take the sample of the private company chairman who is a CPC member as the processing group, and use the PSM method to select the control group in the non-CPC chairman private company, and re-examine the previous research conclusions. The specific processes are as follows. (1) A sample of 3,620 companies whose chairman is a CPC member from 2016 to 2020 is used as the processing group. (2) Whether the chairman is a CPC member is taken as the indicator variable, corporation size (*Size*), financial leverage (*Lev*), profitability (*Roa*), Asset Operations (*OC*), company growth (*Growth*) and age of market (*FirmAge*) are used as matching variables, using non-returning proximity matching to obtain 1,810 company annual samples as Control group. (3) Re-examine the previous research conclusions with annual samples of 1,810 companies in the processing group and the control group. To sum up, the research conclusions obtained after using the PSM matching samples are consistent with previous conclusions.

**Table 8:** Results of the PSM robustness test.

Variable	*PA_D*	*PA_A*	*PA_D*			*PA_A*		
	Full sample	Full sample	Full sample	Party = 1	Party = 0	Full sample	Party = 1	Party = 0
	(1)	(2)	(3)	(4)	(5)	(6)	(7)	(8)
*CCPC*	0.3225***	1.0869***	0.0359	0.2917***	-0.0909	0.1116	0.9675***	-0.3657
	(4.3546)	(4.4660)	(0.1967)	(3.4700)	(-0.4591)	(0.1840)	(3.5277)	(-0.6085)
CCPC*Party			0.2470			0.8323		
			(1.2449)			(1.2649)		
*Party*			0.3513***			1.2146***		
			(3.7108)			(3.9931)		
*Age*	0.2960	1.0894	0.3153	0.1973	0.6442	1.1473	0.6696	2.7347*
	(1.2889)	(1.4320)	(1.3735)	(0.7480)	(1.2475)	(1.5137)	(0.7688)	(1.8040)
*Gender*	-0.3751***	-1.0373**	-0.4047***	-0.3351**	-0.5967**	-1.1288**	-1.0393*	-1.3667*
	(-2.7304)	(-2.2893)	(-2.9204)	(-1.9964)	(-2.5296)	(-2.4985)	(-1.8927)	(-1.7754)
*Edu*	0.0479	0.1777	0.0431	0.0575	-0.0122	0.1603	0.2011	0.0104
	(1.0908)	(1.2375)	(0.9808)	(1.1186)	(-0.1351)	(1.1203)	(1.1942)	(0.0393)
*Share*	-0.6285**	-1.7476**	-0.6045**	-0.4591	-0.8287	-1.6448*	-1.0924	-2.4709
	(-2.3355)	(-1.9894)	(-2.2379)	(-1.4168)	(-1.5387)	(-1.8790)	(-1.0409)	(-1.5699)
*Dual*	-0.0201	-0.0683	-0.0142	0.1552*	-0.5710***	-0.0512	0.4924*	-1.6969***
	(-0.2672)	(-0.2757)	(-0.1887)	(1.7556)	(-3.5241)	(-0.2074)	(1.6958)	(-3.6918)
*Size*	-0.0354	0.3281***	-0.0474	-0.0276	-0.0405	0.2870**	0.3724***	0.2511
	(-0.9492)	(2.6873)	(-1.2655)	(-0.6307)	(-0.5174)	(2.3538)	(2.6049)	(1.0963)
*Lev*	0.1523	0.6483	0.1556	0.0674	0.4820	0.6687	0.3990	1.6455
	(0.6041)	(0.7863)	(0.6153)	(0.2229)	(0.9263)	(0.8142)	(0.4081)	(1.0899)
*Roa*	0.2429	1.6269	0.1612	-0.6991	1.9876	1.3462	-0.9375	5.7766*
	(0.4328)	(0.8957)	(0.2871)	(-0.9908)	(1.6303)	(0.7437)	(-0.4159)	(1.9220)
*OC*	-0.1470	-0.4123	-0.1432	-0.1149	-0.3932*	-0.3934	-0.1983	-1.3552**
	(-1.5149)	(-1.3025)	(-1.4695)	(-0.9992)	(-1.8687)	(-1.2476)	(-0.5269)	(-2.2879)
*Growth*	0.1136	0.2404	0.1236	0.1721	0.0439	0.2686	0.4248	-0.0450
	(1.2973)	(0.8523)	(1.4004)	(1.5807)	(0.2749)	(0.9551)	(1.2117)	(-0.0986)
*First*	0.0537	0.5039	0.0060	-0.0583	0.3871	0.3316	0.0395	1.8916
	(0.1994)	(0.5708)	(0.0220)	(-0.1885)	(0.6500)	(0.3768)	(0.0389)	(1.0800)
*Inde*	-0.0653	-0.3723	0.1083	0.4763	-0.6836	0.2280	1.8551	-2.0477
	(-0.0954)	(-0.1641)	(0.1574)	(0.5811)	(-0.4735)	(0.1007)	(0.6912)	(-0.4929)
*FirmAge*	-0.2950**	-1.1322***	-0.3261***	-0.2055	-0.9474***	-1.2376***	-0.8765*	-2.8707***
	(-2.4988)	(-2.9140)	(-2.7602)	(-1.4818)	(-3.6863)	(-3.1924)	(-1.9217)	(-3.9198)
*_cons*	0.6336	-0.8812	0.6556	0.3316	2.1859	-0.7891	-1.7212	1.1950
	(0.4864)	(-0.2046)	(0.5014)	(0.2214)	(0.7458)	(-0.1839)	(-0.3488)	(0.1376)
*Year*	Control	Control	Control	Control	Control	Control	Control	Control
*Industry*	Control	Control	Control	Control	Control	Control	Control	Control
*Area*	Control	Control	Control	Control	Control	Control	Control	Control
*Observations*	3620	3620	3620	2695	925	3620	2695	925
*Pseudo R2*	0.0399	0.0097	0.0449	0.0453	0.0742	0.0109	0.0107	0.0182
*Wald Chi2/LR Chi2*	183.83	237.02	203.38	154.71	88.53	265.05	195.01	111.23

### 6.4. Endogenous problems

There is also a potential endogeneity that the likelihood of firms having a chairman with or without the CPC status may be explained by firm characteristics. In order to deal with this problem, referring Larcker and Rusticus (2010) [33], Impact Threshold for a Confounding Variable (denoted as ITCV) is used to measure whether the endogeneity problem changes the regression results. If the endogeneity problem is not severe enough to affect the direction and significance of the OLS regression results, the problem can be ignored and the regression results are considered robust. ITCV is measured by the bias correlation between the dependent variable and unobservable variables multiplied by the bias correlation between the independent variable and unobservable variables, and is also the minimum value at which the significance of the results is altered. Exceeding the ITCV indicates that the endogeneity problem is severe enough to alter the regression results. Higher ITCV values indicate that the OLS results are more robust.

As shown in Table 9, column (2) and (6) represents the regression results. The ITCV value in column (2) is 0.045, implying that once the biased correlation between *PA_D* and unobservable variables reach 0. 21(0. 0.045^1/2), the significance of the OLS estimates changes. In addition, according to the coefficients in column (3) and (4), none of them is more than 0.21. Moreover, the ITCV value in column (6) is 0.0496, implying that once the biased correlation between *PA_A* and unobservable variables reach 0.22(0.0496^1/2), the significance of the OLS estimates changes. In addition, according to the coefficients in column (7) and (8), none of them is more than 0.22. Therefore, it can be concluded that the findings of this paper are robust.

**Table 9:** The impact of unobservable confounding variables.

	*PA_D*				*PA_A*			
	(1)	(2)	(3)	(4)	(5)	(6)	(7)	(8)
	*Coefficient*(*P-Value*)	*ITCV*	*Impact*	***Impact*** *raw*	*Coefficient*(*P-Value*)	*ITCV*	*Impact*	*Impact raw*
*CCPC*	0.3616***(0.000)	0.0450			0.7836***(0.000)	0.0496		
*Age*	0.2678(0.157)		0.0021	0.0035	0.4777(0.174)		0.0022	0.0038
*Gender*	-0.2597**(0.011)		-0.0005	-0.0006	-0.4841**(0.023)		-0.0005	-0.0005
*Edu*	0.794**(0.020)		-0.0003	-0.0005	0.1564**(0.014)		-0.0001	-0.0006
*Share*	-0.4258**(0.036)		0.0021	0.0072	-0.8907**(0.018)		0.0021	0.0077
*Dual*	0.0275(0.642)		-0.0001	0.0029	0.0593(0.596)		-0.0001	0.0031
*Size*	0.3876***(0.000)		0.0096	0.0226	0.9939***(0.000)		0.0115	0.0267
*Lev*	-0.1295(0.447)		-0.0001	0.0027	-0.3274(0.318)		-0.0001	0.0034
*Roa*	2.8575***(0.000)		0.0014	0.0017	3.8749***(0.000)		0.0014	0.0018
*OC*	0.1309*(0.062)		-0.0001	0.0002	0.2976**(0.035)		-0.0001	0.0002
*Growth*	-0.2467***(0.000)		0.0005	0.0002	-0.4811***(0.000)		0.0006	0.0002
*First*	0.0107(0.960)		0.0004	0.0006	0.4247(0.307)		0.0005	0.0008
*Inde*	-0.2933(0.581)		0.0002	0.0018	-0.2603(0.798)		0.0002	0.0020
*FirmAge*	0.4497***(0.000)		0.0044	0.0089	0.7929***(0.000)		0.0040	0.0087

## 7. Conclusions and implications

This paper investigates the influence of the chairman’s CPC membership on targeted poverty alleviation based on the hand-collected data of chairman’s CPC membership and the data of A-share listed private companies from 2016 to 2020. The research finds out the chairman of a board as a CPC member not only strengthens the willingness of private companies to participate in targeted poverty alleviation, but also increases the amount of investment in targeted poverty alleviation by private companies. Moreover, the establishment of the CPC branch is an important channel for the chairman’s CPC member status to promote private companies to fulfill the responsibility of targeted poverty alleviation, and the construction of the CPC organization of private companies can strengthen the role of the chairman’s CPC member status in promoting targeted poverty alleviation. In addition, the above research conclusions are still valid under replacing the explained and explanatory variable, and PSM paired sample alleviating endogeneity.

The great success of targeted poverty alleviation is not the end of poverty alleviation, but a new beginning. For a long time in the future, poverty alleviation will become one of the important social responsibilities of listed companies. Therefore, this paper is of significance to consolidate and expand the achievements of poverty alleviation and rural revitalization. And for the government, private companies have developed rapidly and become an important part of the Socialist economy with Chinese characteristics. It is necessary to attract more outstanding private entrepreneurs to join the CPC. Moreover, the construction of the CPC organization of private companies will be greatly accelerated to unleash maximum advantage of socialist market economy. Finally, for private companies, it is necessary to strengthen the construction of Party organizations, give play to the advanced role of CPC members, strive to fulfill social responsibilities, and continue to contribute to the cause of poverty alleviation.
